# Spectral Analysis of Two Coupled Diatomic Rotor Molecules

**DOI:** 10.3390/ijms151119662

**Published:** 2014-10-28

**Authors:** Horace T. Crogman, William G. Harter

**Affiliations:** 1 The Institute for Effective Thinking, Riverside, CA 95340, USA; 2 Department of Physics, University of Arkansas, Fayetteville, AR 72701, USA; E-Mail: wharter@uark.edu

**Keywords:** symmetry, molecular dynamics, BOA, coupled rotors

## Abstract

In a previous article the theory of frame transformation relation between Body Oriented Angular (BOA) states and Lab Weakly Coupled states (LWC) was developed to investigate simple rotor–rotor interactions. By analyzing the quantum spectrum for two coupled diatomic molecules and comparing it with spectrum and probability distribution of simple models, evidence was found that, as we move from a LWC state to a strongly coupled state, a single rotor emerges in the strong limit. In the low coupling, the spectrum was quadratic which indicates the degree of floppiness in the rotor–rotor system. However in the high coupling behavior it was found that the spectrum was linear which corresponds to a rotor deep in a well.

## 1. Introduction

To advance scientific theory it helps to revisit the building blocks. V.H. Van Vleck’s description of angular momentum coupling [1], building on the work of Klien and Casimir [2,3], showed how hyperfine structures arise from a coupling of the electron and nuclear spin. Eckart [4] extended the work of Casimir and others to derive what is now called the Eckart frame, which provides the richness of frame transformations.

However, the Eckart frame was only really useful for small amplitude nuclear motion. Chang and Fano [5] extended the theory to large amplitude motion for a diatomic rotor-electron interaction and indicated the importance of the body and laboratory transformation. They used a phase amplitude procedure to convert the Schrödinger equation to a Voltera-type integral form, which is reduced to a Born-approximation when the long-range interactions are weak. Harter, Patterson, and de Paixao [6] extended Chang and Fano’s electron-diatomic model to treat electron-polyatomic interactions, and showed how Chang and Fano’s frame relations smoothly transform between weakly coupled electron–rotor scattering and tightly bound states of a Born–Oppenheimer Approximation. Our previous work [7] extended frame transformation relations to include rotor–rotor systems. This paper provides evidence that a single composite rotor emerges in the strongly coupled limit developed in [7,8,9,10] and describes the dynamics of floppiness in the weakly coupled limit.

Hougen, Ortigoso, and Kleiner [11,12] treated the K-rotational labeling of eigenstates from an internal rotor system so as to identify good and bad quantum numbers as it moves between the principle of axis method (PAM defined in [12,13]) and the rho axis method (RAM defined in [11,13]). This may be related to labeling schemes in [7], wherein the frame transformation affects the validity of quantum numbers. A growing number of works deal with rotor–rotor systems including that of Hougen and colleagues [8,9], which examined a rotor coupled to a pinwheel, and Groiner [14] who considered a rotor coupled to two pinwheels. We hope to relate the present work to that of Hougen and Kleiner [11] and Groiner’s [14], as well as to the general problems of floppy molecular interactions.

Lemouchi *et al*. [15] reported that the H–H contact of BCO rotor–rotor interactions were shortened as result of the rotational motions that were correlated. They further showed that the lower energy barrier is assigned to a well-correlated synchronous motion between the two adjacent rotors, a point we have made previously [7]. Thus rotor–rotor interactions are important in gas systems, which suggests the timeliness of our approach. Further, the techniques described in this article were used in Crogman *et al*. [9] to investigate floppiness in SF_5_CF_3,_ and it was found that the states below barrier form a band, revealing that the SF_5_CF_3_ molecule has only one degree of freedom below the barrier. Chrysos *et al*. [16] recently reported that they were able to give an interpretation of collision-induced absorption (CIA) and scattering (CIS) by CO_2_–CO_2_ without resorting to short-range interactions to offset the discrepancies between theory and experiment. Another system that could be investigated with this technique is that of methyl nitrate (CF_3_ONO). This complex exists into two different conformers: *cis* and *trans*. The cis conformer has a high internal rotation potential barrier (731 cm^−1^), whereas the *trans* conformer has a low rotational barrier (10 cm^−1^), which can be described in the two bases described in reference [7] as detailed below.

Our work is based on the frame transformation between two bases, namely the Lab Weakly Coupled (LWC) and Body Oriented Angular (BOA) momentum. Note, “BOA” is used to refer the Born–Oppenheimer Approximation, as is explained in [7]. The energy eigenvalues calculated for the multi-pole interaction potential provides insight into the effects of rotor–rotor interactions where strong coupling forms a third frame in which the total angular momentum is conserved along with body quantum numbers *n*_l_, *n*_R_ and *K*.

## 2. Result and Discussion

**Figure 1:**
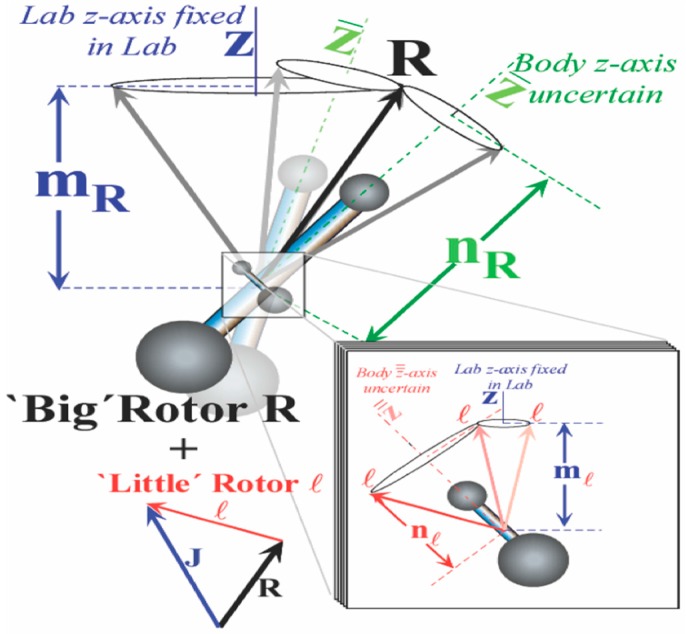
Schematic of coupled rotor system taken from [7]. The angular momentum *J*, *R*, and *ℓ* are shown along with their projection of the lab and body axis.

Previously, we considered interactions involving angular momentum [7]. Here, we introduce an angular coordinate interaction potential that will determine the floppiness of two rotors. A two-rotors model is sketched in Figure 1. Before coupling we imagine that a “big” rotor R is oriented at some angle β relative to the lab while the other “little” rotor axis is oriented at lab polar angle θ, and the angle Θ lies between them (if the two were in the same plane with Θ = β ± θ as shown in [7,8,9,10]). We use R to designate the “big” rotor momentum quanta, while *ℓ* designates that of the “little” or “faster” rotor. (The formalism must be adapted to the rotor being identical as well as to having the R rotor being “faster or smaller”).

We consider a model such that the two rotors are connected to each other by a spring. We use Equation (1) to show a simple way to model this behavior.


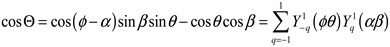
(1)

To further restrict the floppiness of the rotor system, we add higher powers of cosine. We write a general equation of the expanding of the powers of cosine in terms of spherical harmonics. The scalar potential interaction is a function of the cosine angle of Θ as given in formulas (Equations (2)–(4)).
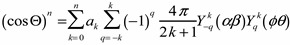
(2)where [17] shows that *a_k_* is such that:
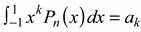
(3)

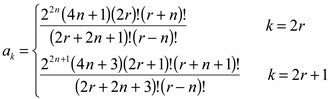
(4)

For simplicity of modeling, we will assume a general interaction of the form as in Equation (5a):
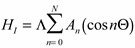
(5a)where *cosn*Θ can be expressed as Chebyshev polynomial of the first kind as in Equation (5b).
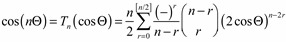
(5b)

Therefore Equation (6) is the general combination Hamiltonian of the following form:
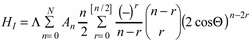
(6)where is Λ the coupling constant. We represent the fully coupled symmetric rotor Hamiltonian as:
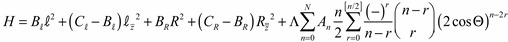
(7)

**Figure 2:**
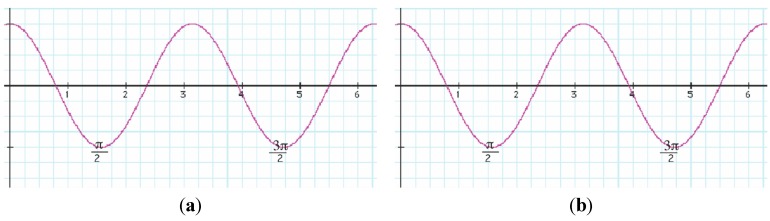
The graph of cos*n*Θ (**a**) *n* = 2 and (**b**) *n* = 3.

By varying Λ in Equation (7), we move from the LWC to the regime where the coupling is very strong. The truncated Hamiltonian matrix is calculated for various values of Λ for *N* = 2. We chose *N* = 2 based on the fact that *cosn*Θ has a minimum at Θ = π/2 as shown in Figure 2. The strong coupling limit is where the two rotors would be expected to lock perpendicularly to each other, thus forming an oblate top.

The general procedure is to diagonalize the Hamiltonian for the rotor–rotor interaction at the different coupling strengths and then weave our way through the energy spectrum looking for the regions of a composite rotor’s top behavior.

The matrix element Equation (8) is calculated using an LWC framework. We apply the Wigner–Eckart theorem to our Hamiltonian since it has the form 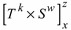 where *z* and *x* are zero. Consequently, the general matrix element of a scalar potential [18,19,20] is given by the following:
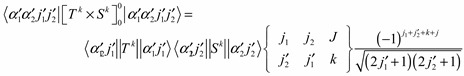
(8a)

Thus the explicit matrix elements for the combination Hamiltonian are:
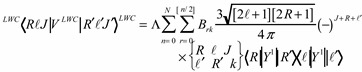
(8b)where:
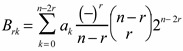
(8c)

The expression in the LWC basis is the product of a Racah coefficient [18,19,20] with the reduced matrix elements of the coupled system, which is simpler than the expression in the BOA involving multiple Clebsch–Gordan coefficients Equation (9). As a result it is more convenient to program in LWC basis and then transform to the BOA basis for the sake of computational time. From a mathematical standpoint, the higher powers of cosine do not change the form of Equation (8b) except that *k* has a value corresponding to the particular power, but the result may be quite different physically.
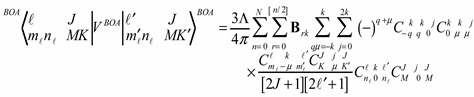
(9)

To illustrate the behavior of our coupling Hamiltonian we will now consider the case where two diatomic molecules are coupled together.

### 2.1. Example: Coupling between Two Diatomic Molecules X_2_ with n = 2

**Figure 3:**
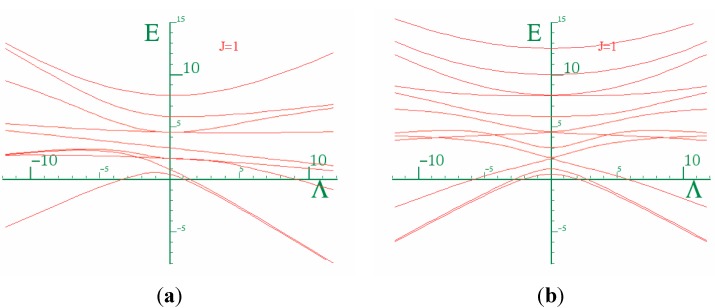
Energy *vs.* coupling of X_2_ for *J* = 1. (**a**) *n* = 2; (**b**) *n* = 3.

Let us consider two X_2_ rotors interacting through a periodic potential as given by Equation (5). Figure 3a,b shows the energy E *vs.* the coupling constant Λ. We choose B = 0.5 kg·m^−2^ as the rotational constant to approximate that of H_2_ given in [21] for 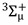. This value was rescaled by a factor of 10 on the plot. The plots of Figure 3a,b correspond to the interaction potential in Figure 2 with *n* = 2 and 3 respectively.

We know that all three angular momenta are good quantum numbers in the LWC basis. However, in the BOA basis only angular momenta *ℓ* and *J* remain so whereas, for higher terms in the multi-pole expansion, the *ℓ* quantum becomes uncertain and the higher *ℓ* states must be coupled according to Equation (8b). As a result, *ℓ* will not be a good quantum number even though J is always conserved in the absence of external forces. To achieve conservation, the angle between the two rotors is more nearly conserved with the higher powers of the cosine. Figure 3 shows that as we increase the power of cosine, there is mixing of higher *ℓ* levels. Due to lab symmetry (absence of external torque) the matrix for Equation (8) is a block diagonal in *J* due to the conservation of total angular momentum. However, there is a multipolarity-k-dependent mixing of the levels and *ℓ* that is not conserved. Physically, the vibration or flopping is reduced and the rotor of angular momentum *ℓ* acts like a passenger on the rotor of the angular momentum R with neither *ℓ* nor R being constant. When the rotors are locked in together, the only degrees of freedom remaining are those of the individual rotors’ spin *n_ℓ_* or *n_R_* about their body axis. A system in which one of the rotors is allowed to move in this fashion is discussed in [11], and the case where both are allowed to turn is studied in [22].

**Figure 4:**
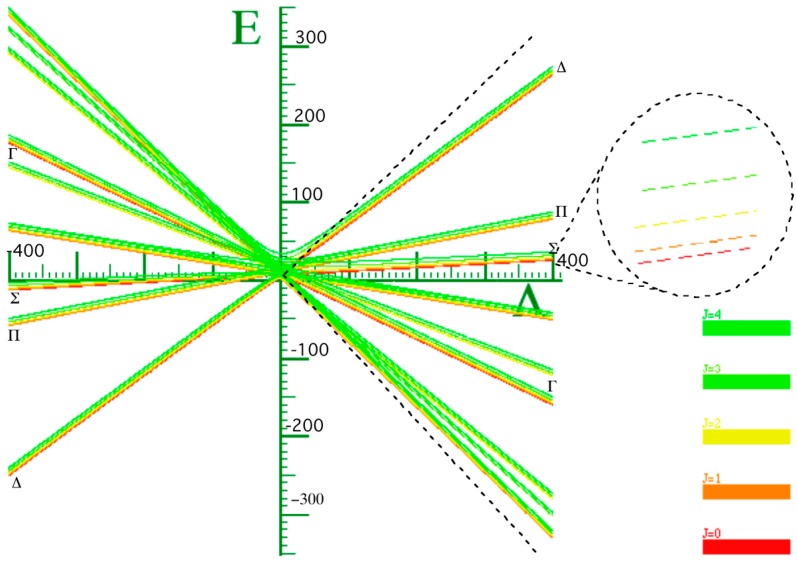
Energy *vs.* coupling of X_2_ molecule for *J* = 1, *n* = 2.

To really make a case for a single composite rotor emerging from this coupled rotor system, we must turn coupling Λ to the highest possible value. In light of Figure 3 we see that at low coupling of Λ, the eigen-energies are unsettled. As coupling increases, the levels come together to form bands. In Figure 4, the energy band label (Ʃ) corresponds to the angular momentum values of 0, 1, 2, 3, and 4. The next band of levels (Π) contains only four levels with values 1, 2, 3, and 4. The next band is partly a duplication of the first. The spacing between the energy levels in some of the bands follows the Lande interval rule as presented in Table 1.

**Table 1:** The energy spacing values ∆E_j_.

Ʃ Band	∆ Band	Γ Band
1.07784	1.0337	1.0101
2.15526	2.0675	2.0204
3.2361	3.101	3.0304
4.3029	4.1348	4.04004

Figure 4 is a plot of two X_2_ molecules interacting through a cosine potential with *n* = 2. The inertia coefficient around the *B* axis was chosen to be 0.5 kg^−1^·m^−2^. The coefficient around the *C* axis is infinitely large for a diatomic molecule, but this does not contribute to the energy since there is no spin or twist around this axis. The coupled system has an overall B coefficient when it is locked together with 0.5 kg^−1^·m^−2^. From the Lande interval, we get an overall B coefficient of 0.5 kg^−1^·m^−2^. In Table 1, the energy spacing ∆*E_j_ = E_j_ − E_j−1_* follows the *ℓ* interval rule.

The graph in Figure 3a is asymmetric while the graph in Figure 3b displays a greater degree of symmetry. The reason is that when *n* = 3 when there are three minima, two at 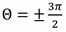 (one on either side of Θ = *π*) and one at Θ = *π*. Therefore, the graph is symmetric at about π. This is a stable point. For *n* having odd values, the graphs will be symmetric around π. Thus the two diatomic molecules would prefer aligning parallel to each other at Θ = *π* for *N* = 3 but have a perpendicular alignment for 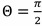, which corresponds to *N* = 2.

The wavefunctions for a free diatomic molecule are the spherical harmonics. We use Clebsch–Gordan’s coefficient to couple two or more diatomic molecules together. A very strong coupling constant would take us to the BOA basis. The BOA wavefunction derived in [7] is used to describe the coupled system of two diatomic molecules in the strong limit with body quantum number *n_ℓ_* = 0 and *n_R_* = 0 required for two diatomic molecules. Thus our BOA wavefunction reduces to the wavefunction described in [6,7].


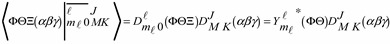
(10)

**Figure 5:**
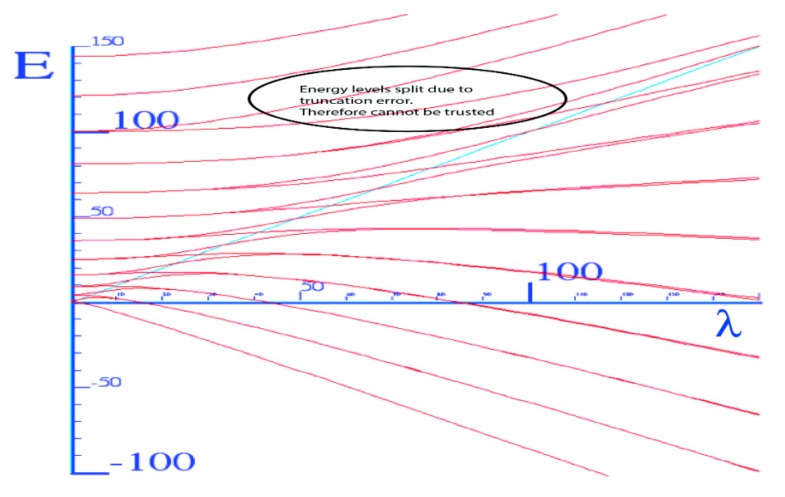
Energy *vs.* coupling for the Mathieu (truncation errors above *E* = 100 cm^−1^).

**Figure 6:**
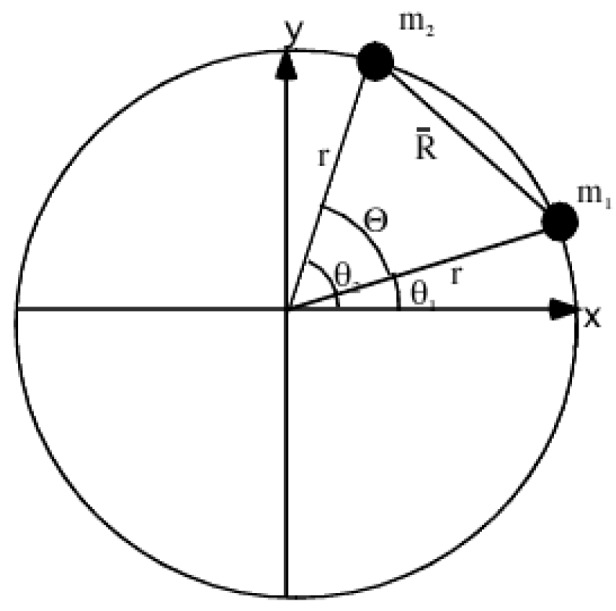
Illustration of two particles moving on a ring.

**Figure 7:**
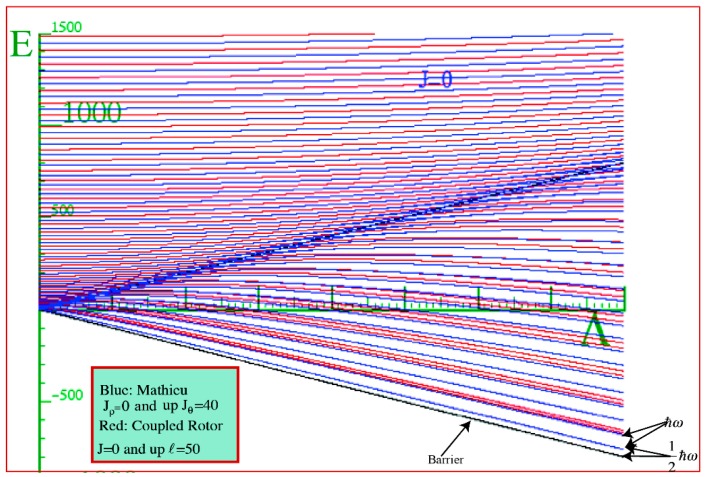
The comparison of the energy behavior above and behavior with the coupling constant between the two rotors model and the super-Mathieu for *J* = 0 and *ℓ* = 50.

**Figure 8:**
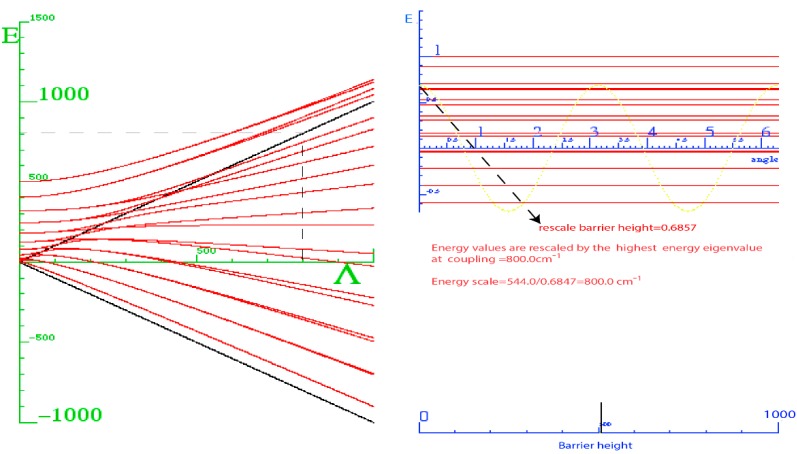
**Left**: Energy *vs.* coupling constant for Equation (21) with *J*_Θ_ = 10 *and B_ℓ_* = *B_R_* = 2.5 *cm^−1^*; **Right**: Displaying the potential energy curve and energy levels at single coupling constant Λ = 800 cm^−1^ and barrier height = 540 cm^−1^.

**Figure 9:**
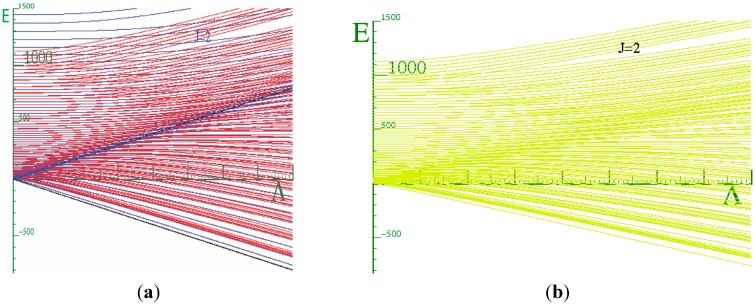
(**a**) Energy Spectrum for the two coupled rotor models super-imposed on the super-Mathieu for *J* = 2; (**b**) Energy Spectrum for the two coupled rotor models for *J* = 2 display alone.

**Figure 10:**
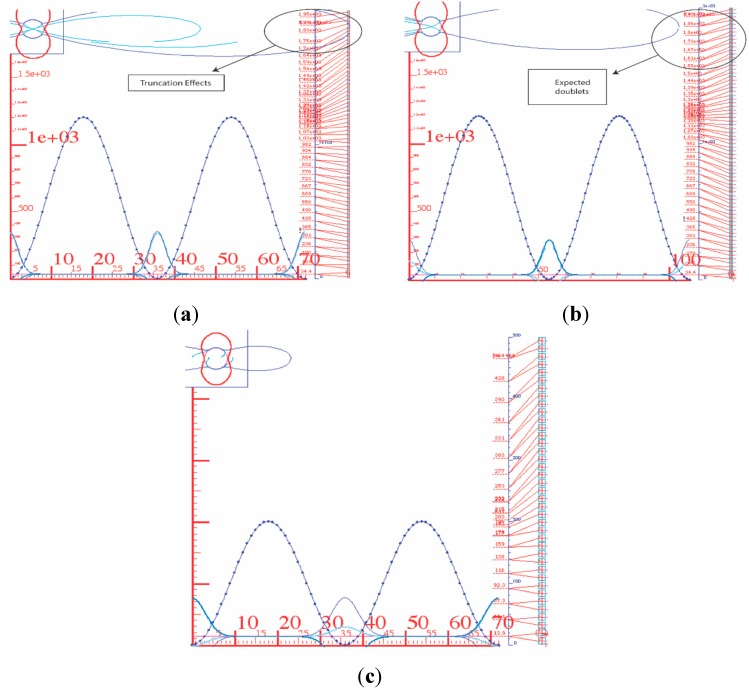
Energy level diagrams. (**a**) High-energy truncation effects occur for a high barrier (*V* = 1200 cm^−1^) and a low truncation value (*J_Θ_* = 36); (**b**) High-energy truncation effects reduced for higher truncation values (*J_Θ_* = 54); (**c**) Effects on truncation effects reduced for (*V* = 200 cm^−1^).

Now, Equation (10) presents the picture of an electron interacting with a bare diatomic rotor molecule [6]. The BOA basis corresponds to a third body emerging from joining two rotor systems, which can also be seen in our previous work [7,8,9,10]. Figure 4 of [9] provides a schematic visual of composite rotor forms in SF_5_CF_3_, and in Figure 4, Figure 5, Figure 6, Figure 7, Figure 8, Figure 9, Figure 10 and Figure 11 of [8] we are given a visual in the context of rotational energy (RES).

The uncertainty of both R and *ℓ* angular momenta results in localizing our wavefunction in the BOA basis, which is approximately given by a single wavefunction of total angular momentum *J*. In other words, the rotors are rigidly connected to each other and are not able to flop around. We coin the term “extremely constricted BOA state” for the situation where the *ℓ* quantum number becomes uncertain. The following simple sum *ℓ* constituent is an approximation of the BOA constricted state. Equation (11) reveals the composite rotor as these rotors become extremely constricted.


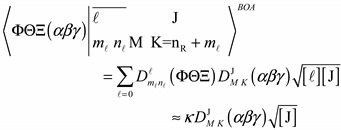
(11)

However, the foregoing analysis constitutes a “shot-gun” wedding of the two rotors by Hamiltonian Equation (6), and it is still unclear whether the marriage is a success. To help our understanding, we turn to simpler models for which the diagonalization is less time consuming.

### 2.2. Comparative Studies with a 3D-Coupled Rotor System

#### 2.2.1. A Free 1D Rotor Interacting Cosine Potential

We construct a simple model to understand the mechanism by which a single composite rotor emerges from two coupled rotors. A weakly coupled system or floppy system is related to the opposite situation where they are strongly coupled. To begin, we will illustrate the effects of a free rotor in a Mathieu type potential. Angular momentum is transverse to its body axis. The Hamiltonian for a simple one dimensional free rotor system is given by Equation (12).


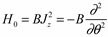
(12)

One may visualize a disk rotating about some imaginary axis perpendicular to the center of the disk. The wavefunction for a state ⎥*m*〉 of definite momentum (*m* = 0, ±1, …,) in *ℏ*-units for such a Hamiltonian is given by Equation (12):

〈*θ*⎥*m*〉 = *e^imθ^* (13)

A quasi-quadratic free 1D rotor spectrum results in,

*H_0_*⎥*m*〉 = *Bm^2^ m*〉 (14)

Now with a cosine potential, the Hamiltonian is represented in the Θ angle basis by a Mathieu type Schrödinger Equation (15a):
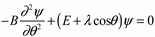
(15a)

The solution of Equation (15a) is complicated since simple angular momentum states ⎥*m*〉 are no longer eigensolutions. Therefore we use a new base in Equation (15b), which is a linear combination of the angular momentum states ⎥*m*〉.


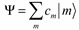
(15b)

The matrix representation of the Hamiltonian in the ⎥*m*〉 basis is an infinite matrix as given in Equation (16):
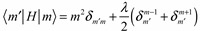
(16)

The infinite matrix in ⎥*m*〉 basis may be truncated to a finite set with *m ≤ m_cutoff_* in order to diagonalize it to give the energy levels. The results depend on the sensitivity of the coupling constant λ, which determines whether or not the rotor is above the barrier. Whether the rotor in state ⎥*ε_k_*〉 is stuck or loose depends on its energy level (eigenvalue *ε_k_*) being above or below the cosine barrier λ.

The graph of energy eigenvalues *vs.* λ (Figure 5) shows that above the barrier the spectrum is quasi-quadratic (nearly free rotors) but below, the barrier of the spectrum is quasi-linear (trapped rotors in oscillator potential). The trapped energy levels below the barrier are nearly doubly degenerated because there are two equivalent wells. Near the barrier, the levels split and recombine with other levels to eventually form doubly degenerated quasi-free rotor levels above the barrier.

#### 2.2.2. Two 1D Rotors Interacting through Angular Potential

A coupled three-dimensional rotor system is considerably more complicated. However, we can be ingenious about using the insight from the one dimensional Mathieu problem. Suppose that both rotors are constrained to rotate about their body axes normal to a plane in which they both lie as illustrated by (Figure 6). We can use a classical mechanic description for two particles on rings or two discs rotating about the same axle at different angular speeds. Equations (17)–(20) is the derivation of the Lagrangian for the system in Figure 6.

*R*^2^ = 2*r*^2^(1 − cos(*θ*_2_ − *θ*_1_)) (17a)

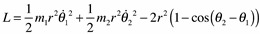
(17b)

Using the center mass frame we have:
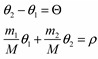
(18)

To quantize our system, it is best to represent our model by using a classical Hamiltonian,
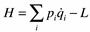
(19a)
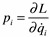
(19b)which gives:
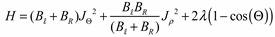
(20)

Our Hamiltonian is in the form of an overall rotation plus the Mathieu equation both of which we have investigated to some degree. Since the angle of the potential between two coupled diatomic rotors has factor 2, we replace Θ with *2*Θ in Equation (20). The constant term 2λ is dropped in the standard form of Mathieu’s Equation to give (21b). The Hamiltonian of free rotation is separated. Its eigenfunctions 〈*ρ*⎥*m_ρ_*〉 = *e^im_ρ^ρ^_^*/2π will be factors in the overall wave 〈*ρ*⎥*m_ρ_* 〉〈Θ⎥*ψ*〉 that depends also on the solution to the internal Mathieu Hamiltonian:
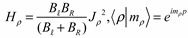
(21a)

*H*_Θ_ = (*B_ℓ_* + *B_R_*)*J*_Θ_^2^ − 2*λ*cos(2Θ), 〈Θ⎥*ψ*〉 = *e*^im_Θ_Θ^ (21b)

The red curves in (Figure 7) are those due to the Hamiltonian Equation (20) of two coupled rotors. The blue curves come as a result of solving the Mathieu Equation (21). The black lines in the figure are the top and bottom of the potential barrier. We expect that at *J* = 0, our simple model will be present in the three-dimensional system.

Our strategy is to supper-impose the blue curves that were obtained by solving Equation (21a) onto the red energy spectrum curves, which are the eigenvalue solutions of Equation (18). In the low coupling limit (LWC) we observe that both sets of energy spectrum were quasi-quadratic above the barrier (Figure 8). This is an indication that our coupled system is loosely correlated or very floppy. While in the high limit (BOA), that is below the barrier, we found that the levels come together to form doublets and exhibit near linear behavior between the relative spacing of these doublets in both the simple and 3D-coupled systems. Our Hamiltonian is a function of angular *ℓ*, *R* and *J*. For the plot shown in Figure 7, *J* = 0, *ℓ* = 50 and *R* = ⎥*𝐽* − *ℓ*⎥. Thus the number of states considered was 51. If we do not include enough states, the red curves would curve away as the barrier is approaching from the left to give a similar behavior as the blue curves above the barrier shown in Figure 7. At low energy, the doublets squeeze tighter together. This is strong evidence that the rotors are becoming locked together to form a single composite rotor.

#### 2.2.3. Coupling Behavior of the Simple Rotor Model with the Full 3D Model of Two Coupled Rotor-Systems

We are eluded by the fact that there is not a match for the lower energy of the simple model with that of the coupled system. Oftentimes a shift comes about in matrix equation if the diagonal terms are changing. As a result, we investigated the interaction term of our rotor-coupled system and found that there are terms on the diagonal that are due to the coupling (this behavior is due to the centrifugal distortion). In comparison to the interaction of the Mathieu equation, there are no such terms in the simple model.

Consequently, we now introduce such a term into the Mathieu equation to do just that. When we first wrote down the Hamiltonian for two particles moving on a ring, a term resulted in on-diagonal terms due to the interaction, which was discarded. Putting this term back caused the Mathieu curves to shift too far upwards. Thus we introduced a fractional parameter (*b*) to control the shift.

*H* = (*B_ℓ_* + *B_R_*)*J*_Θ_^2^ + *λ*(*b* − cos2(Θ)) (22)

When *b* = 0.125, a more realistic match is reproduced between the Mathieu and the two-coupled rotor system. However as we increase the coupling to value greater than 800 there is a noticeable mismatch between the lowest energy spectra of the blue curves with that of the red curves. This is an indication that the shift was not a result of the absence of the on-diagonal term in the simple model. Yet, another way to understand the shift is to change the inertia constant in our simple model. There seems to be a match in the lines when the inertia is eight times the original. However, this does not seem to be the mechanism for the shift since most of the other lines are off. The explanation for this shift is that simple Mathieu is 1D but the coupled system has a higher degree of freedom.

In the Mathieu model, the first level matches the zero point of a 1D harmonic oscillator, whereas the lowest level for *J* = 0 in the coupled system corresponds to a 3D oscillator. We will next consider higher Js and observe what happens there.

We solved the Hamiltonian for *J* = 1, 2, and 3 and found that there is a perfect correspondence between the lowest energy of the Mathieu system and the coupled rotor system (Figure 9) for *J* ≥ 2. There is a strong indication that our system becomes locked as we go to very high coupling constants. We observed a quadratic behavior above the barrier, which is an indication of the degree of floppiness in our system. A linear behavior in the energy was observed as illustrated by (Figure 8). This corresponds to a system that is extremely correlated or locked. When this happens, the system moves together as one unit while vibrating rapidly.

### 2.3. Truncation Effects in a Coupled Rotor System

In Figure 5, the lower energy states are doubly degenerated, but they split near the top of the barrier. However, they recombine into different doubly degenerated energy levels as the coupling constant increases. For higher energy states they may fail to recombine as a result of truncation of our matrix. The circled region of Figure 5 shows splitting that arises from truncation above the barrier. To prevent this anti-fact we must introduce more base states. The energy level diagrams in Figure 10 illustrate truncation effects, where the top states are splitting apart. With the potential barrier in Figure 10 being large (that is, comparable with the largest energy level) and *J_Θ_* = 36, only the levels below the barrier height may be trusted since those above the barrier are no longer expected to be quadratic doublets.

Figure 10b, with a higher truncation value (*J_Θ_* = 54), shows the expected quadratic behavior above the barrier. Note that when the top of the barrier is much lower (*J_Θ_* = 36) (Figure 10c), then we still observe a quadratic spacing above the barrier in spite of low (↓) truncation values. This indicates where the diagonalization may be valid.

The full rotor–rotor diagonalization also shows truncation effects with respect to the cut-off of the *ℓ* values used in the starting basis. It is important to see how truncated *ℓ* affects the calculation. To this end we consider another simple model, the particle orbiting a 2D-cosine potential or “a particle orbiting in a peanut”.

### 2.4. Orbits in 2D-Cosine Potential (“Particle in a Peanut”)

**Figure 11:**
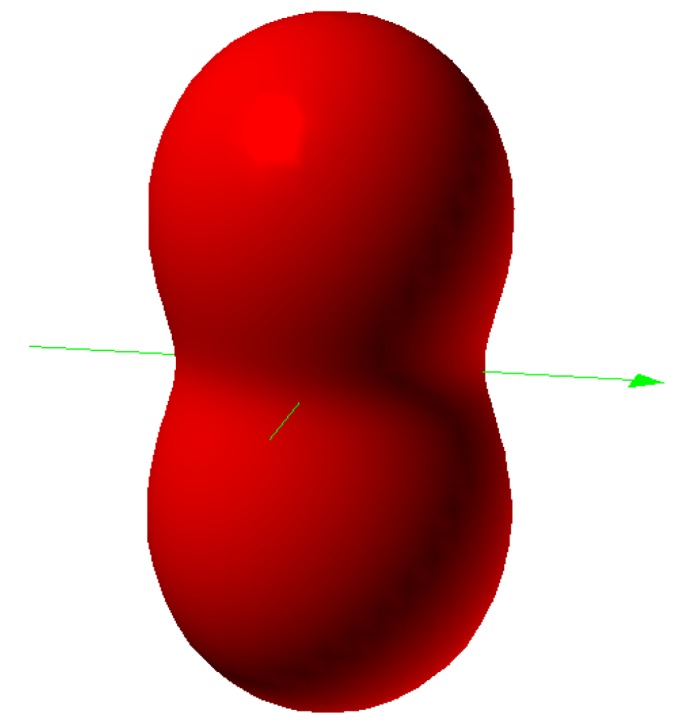
Potential surface of a “peanut”.

Let’s consider a particle constrained to move on a peanut-like potential surface shown in (Figure 11). The Hamiltonian of this model is as follows:

*H* = *Bℓ*^2^ + *λ* cos(2Θ) (23)

The wavefunction is given by superposition of the spherical harmonics ⎥***ℓm***〉 or 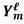.


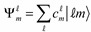
(24)

where 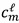 are elements of the eigenvectors after the diagonalizing H. The Hamiltonian matrix Equation (25) to be diagonalized is given by:
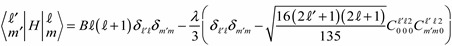
(25)

#### 2.4.1. Probability Distribution Comparison between a Rotor–Rotor Model and a Particle in 2D Cosine Potential

The angular 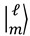 state probability is the square of the elements in the eigenvectors as in Equation (26), that is:
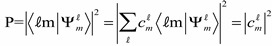
(26)

**Figure 12:**
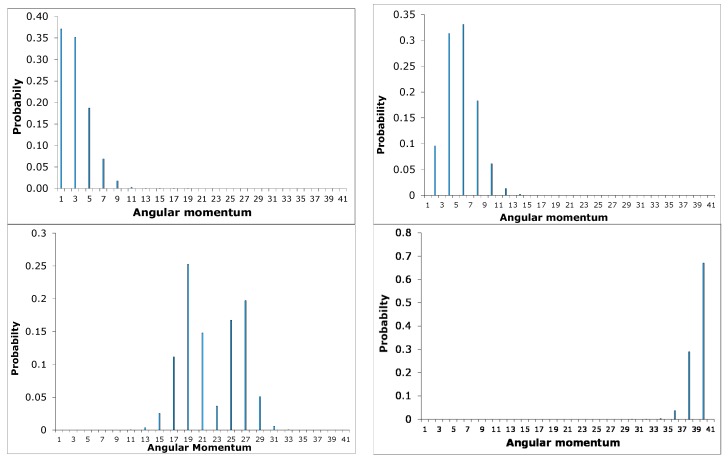
Probability distribution at *m* = 0 for a particle constrained to move on the surface of a “peanut” with *ℓ* = 0 − 40.

Figure 12 shows a plot of probability 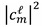 *vs.* *ℓ* for corresponding *E_ℓm_* and *m*-values = 0, 1, 2…

**Figure 13:**
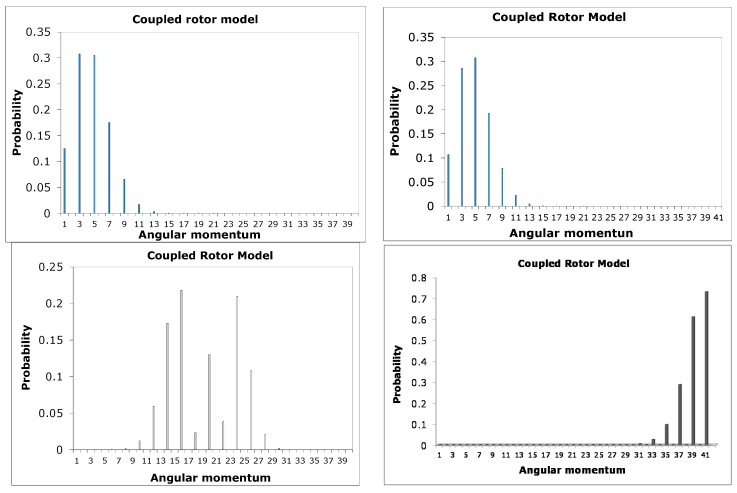
Probability distribution at *J* = 0 for a particle constrained for two coupled rotors *ℓ* = 0 − 40.

We compare *m* = 0 for a single particle constrained to move on the surface of a peanut-like shape with the two coupled rotors for *J* = 0. It was observed that there is good correspondence in the behavior of their probability distribution as illustrated in Figure 12 and Figure 13. They both start out Poissonian, however, as the angular momentum *ℓ* increases their distributions are no longer Poissonian. In other words this behavior in distribution is a non-classical effect.

#### 2.4.2. The Determination of Extremely Strongly Coupled Rotors

As we move from the LWC basis to the BOA basis by increasing the coupling strength, the two rotors eventually become locked together so that they move as a single system. In the LWC model, the *K* quantum number is not good but improves in the BOA region. Our goal here is to find the coupling constant where *K* is good. To do this we must compute the expectation value *K* by using the eigenvector of the coupled system as coupling increases. The expectation value of *K* is given by:
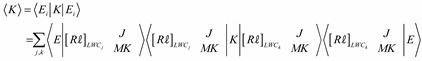
(27)where Equation (27) is a matrix equation that has off-diagonal elements when the coupling is weak. The matrix shown below was computed for *𝐽* = 1, *ℓ* = 0.2 and a coupling constant of about 0.08. It is evident that at such weak interactions, we are not in the BOA basis since Equation (28a) shows off-diagonal terms.


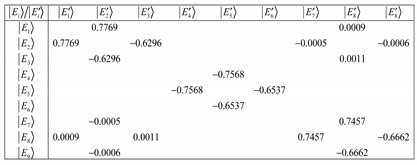
(28a)

On the other hand, when the coupling constant is about 8000 then Equation (28a) reduces to Equation (28b). Increasing the coupling constant continuously gives Equation (28c). However, we have chosen *𝐽* = 1, *ℓ* = 0.2 from which at low *ℓ* values, there will be the issue of truncation error to contend with. The matrix reduces at much lower coupling with a large number of angular momentum *ℓ* states. This suggests that the more angular momentumstates *ℓ* states, the more BOA-constricted are the rotors for high coupling values. Because of truncation errors close to the barrier, we are only interested in the K quantum numbers that correspond to the states deep down.


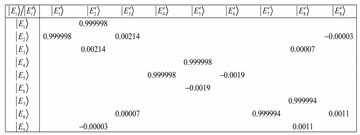
(28b)


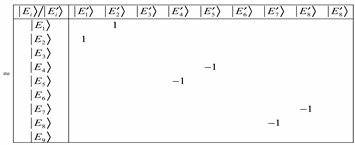
(28c)

We observed that along the diagonal of Equation (28c) there are two-by-two matrices such as:


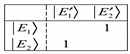
(29)which have eigenvalues of +1 or −1. As a result, the eigenvalues are −1 0 1, −1 0 1, and −1 0 1. These are *K* quantum numbers for *J* = 1. Given that Equation (29) is a B matrix, then the eigenvectors in Equation (30) for the BOA basis are represented by:


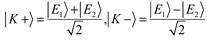
(30)

Once we know at what coupling constant the *K* quantum number is good, then the search for a rotor spectrum can begin. Wilson, *et al*. [23] were the first to treat the effect of centrifugal distortion in rotational energy level by considering the general rotor Hamiltonian,


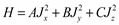
(31)

**Figure 14:**
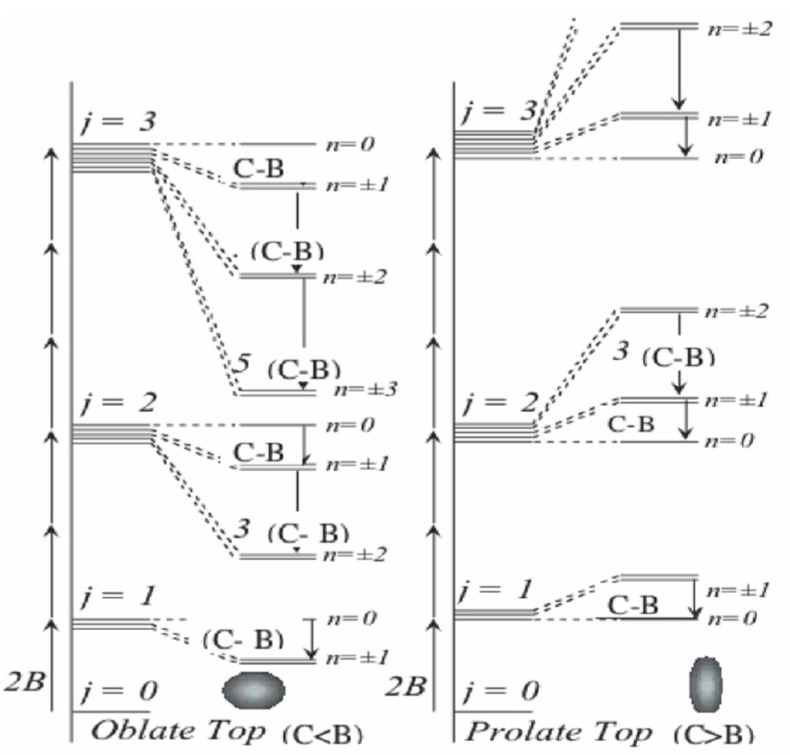
Quantum rotor levels for *J* = 0,1,3,….

**Figure 15:**
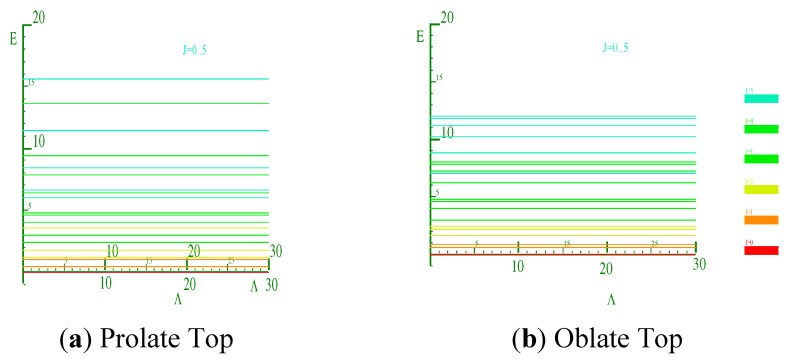
(**a**) and (**b**) show the energy spectrum for a prolate and an oblate molecule respectively.

Equation (31) describes three classes of rotor tops: a prolate top if *A = B < C*, an oblate top if *B = C > A*, and an asymmetric top if *A ≠ B ≠ C*. Thus, we illustrate the energy spectrum for a prolate and an oblate tops in Figure 14 and Figure 15. The goal here is to compare the spectrum of the composite rotor with that of Figure 15.

**Figure 16:**
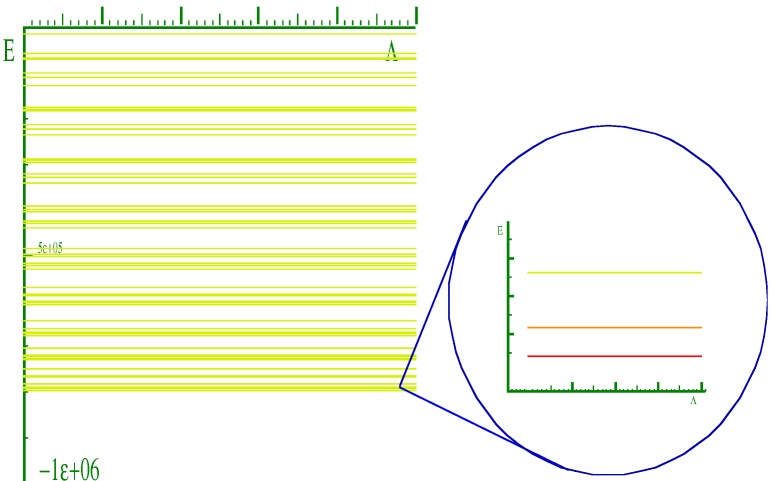
Energy spectrum, calculated for coupling value of 800,000.0 and *J* = 0 − 2 *and ℓ* = 0 − 40. Zoom energy spectrum for the lowest energy band to far right.

**Figure 17:**
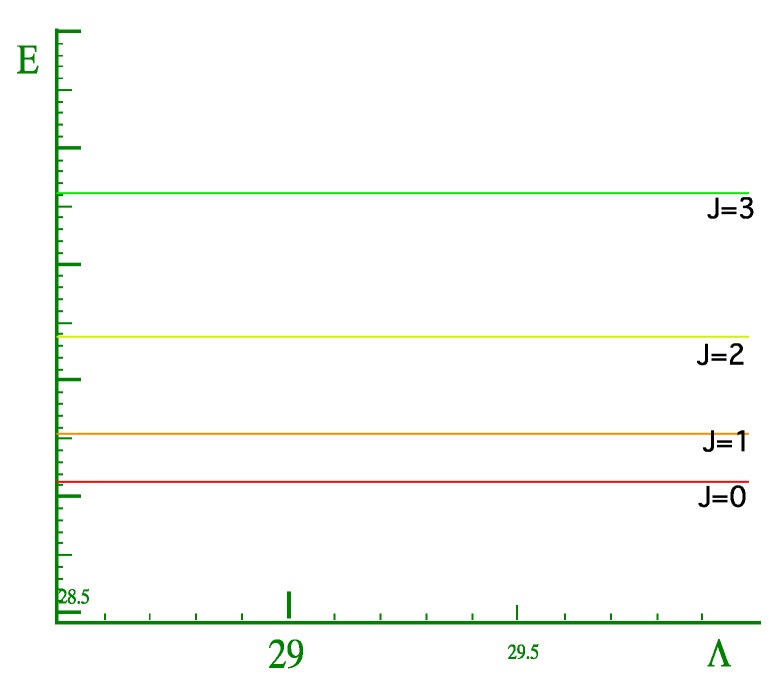
Rotor spectrum for a coupled rotor system for *K* = 0 at the *J* values shown above.

Recall in Section 2, that due to truncation effects, only the states below the barrier should be trusted. Therefore, we began the search for a rotor spectrum at lowest energy levels in the energy spectra of the coupled rotor system as shown by (Figure 16). One should note that a coupling at which the *K* quantum numbers become good increases with *J*. Recall also that in Section 2, for the values of *J* > 1, we observed that the lowest levels do not have *K* = 0. This phenomenon is not clearly understood yet, however, to find a rotor spectrum we must begin the search above these levels or the band of levels where *K* = 0 levels may be found. Figure 17 shows the energy spectrum of the rotor molecule for *K* = 0 which is obtained from the spectrum of the two coupled rotors as shown in Figure 16. Although, we are able to find a spectrum for *K* = 0, we were unsuccessful in getting a match for *K* = 1 or higher values. *K* = 0 does not provide us with enough information to decide whether or not we have a prolate spectrum, an oblate spectrum, or something else.

However, we speculate that our potential does not completely describe the phase space, and as a result the marriage between the two rotors was unsuccessful. Nevertheless, our potential was enough to tell whether or not the system would lock. We did find evidence of locking of the two coupled rotor molecules.

## 3. Conclusions

The two bases studied in this paper are LWC and BOA. The LWC basis corresponds to the system that is weakly coupled, whereas the BOA is the regime of strong coupling. The objective has been to understand rotational relativity. We expected that under strong coupling our rotor system would lock. That is, the strong correlation between two coupled rotors would cause a third composite to emerge. Above the barrier we observed that the energy spectrum is quadratic, which is an indication of the existence of free rotor or floppy type molecules. Below the barrier however, we see that the spectra was very linear thus indicating that the rotors are correlated. The lowest energy spectrum for the case *J* = 0 has doublets. This is our indication that the rotor–rotor coupling is causing the system to move as one body.

We have tried to determine what new rotor molecule was formed but thus far we were only able to observe the spectrum for the *K* = 0 state which gives the correct spacing that would indicate a rigid composite body. We have found a particular coupling value where *K* is a good quantum number. This is an indication that we are in the BOA basis where the coupled system moves as a rigid body. The goodness of *K* changes with increased *J* values and also with truncation effects. To get around the truncation effects we carried out the quantum calculation with large number of states and kept the eigenvectors that corresponded to the lower states. These were used to find the expected value of *K*.

The quantum analysis of two coupled rotor molecules failed to identify the spectrum for values higher than *K* = 0. Crogman *et al*. [10] developed a theory that shows the various symmetry types of composite rotors that emerge from the coupled rotor, and in [8] we used rotational energy surfaces to study the emergence of a single composite molecule from the two coupled rotor molecules. The next step is to consider other coupling schemes and higher *ℓ*-states in the same investigative context.
